# Awareness, Perceptions, Willingness, and Feasibility of mHealth Apps Among People Living With Epilepsy: Cross-Sectional Questionnaire Study

**DOI:** 10.2196/80283

**Published:** 2025-12-10

**Authors:** Bushra Batool Zahra, Muhammad Amir Hamza, Rehana Sarwat, Hina Batool Zahra, Ayesha Azam, Ali Ahmed

**Affiliations:** 1 Department of Pharmacy Practice, Faculty of Pharmaceutical Sciences Riphah International University Islamabad Pakistan; 2 Division of Infectious Diseases and Global Public Health School of Medicine University of California San Diego San Diego, CA United States

**Keywords:** mobile health apps, epilepsy, feasibility, treatment compliance, digital tool, cross-sectional study

## Abstract

**Background:**

The rapid expansion of mobile health (mHealth) apps has transformed health care delivery worldwide. Despite their potential to improve epilepsy care, a substantial treatment gap remains, especially in low- and middle-income countries, due to limited resources, stigma, and low adoption of digital technologies. Although mHealth apps can bridge these disparities, their impact depends on acceptance and use by the target population.

**Objective:**

We aimed to assess the awareness, feasibility, willingness, perception, and factors influencing these behaviors for the usage of mHealth apps among people living with epilepsy in Pakistan.

**Methods:**

We conducted a cross-sectional analytical survey between March and July 2024 among people living with epilepsy attending the Pakistan Institute of Medical Sciences (PIMS). Participants completed a validated, self-administered questionnaire with 33 items across 5 domains. We recruited 406 participants through convenience sampling and analyzed the data using SPSS version 23.0 (IBM Corp). Through multivariable linear regression analysis, we explored factors associated with people living with epilepsy willingness to use mHealth apps. Correlation analysis was used to elucidate the association among awareness, perception, feasibility, and willingness.

**Results:**

Among 406 participants, 53.7% (n=218) were male, 64.5% (n=262) were married, and 89.2% (n=362) were identified as Muslim. Although 86.2% (n=350) of participants have heard about mHealth apps for epilepsy management, 78.1% (n=317) expressed negative perceptions of their use. More than half, 69% (n=280), reported concerns about the privacy of their medical information online, and 78.1% (n=317) were not comfortable using mHealth apps on smartphones or tablets. Multivariable linear regression analysis revealed that rural residents (*P*=.05), those with a college education (*P*<.001), and participants with a treatment duration of 2-3 years (*P*<.001) significantly influenced participants’ willingness. Correlation analysis showed a weak negative relationship between awareness and feasibility (ρ=–0.124; *P*=.01) and a weak positive relationship between awareness and willingness (ρ=0.013; *P*=.07).

**Conclusions:**

To expand mHealth use for epilepsy care in Pakistan, stakeholders must address concerns about digital literacy, data privacy, and trust. Collaborative efforts involving government, technologists, nongovernmental organizations, academia, and health care providers can improve education, enhance data security, and adapt mHealth tools to local needs, ultimately improving treatment access and outcomes for people living with epilepsy.

## Introduction

Epilepsy is one of the chronic neurological diseases that require special medical care due to susceptibility to experiencing epileptic seizures, resulting in neurological, cognitive, psychological, and social implications [1,2]. The global prevalence of active epilepsy, encompassing both idiopathic and secondary forms, is 658 per 100,000 individuals. The Global Burden of Disease Study 2021 Collaborators found that 80% of the bulk of the epilepsy burden was reported in low- and middle-income countries (LMICs) according to World Bank country income classifications [3]. In Pakistan, the prevalence of epilepsy is estimated to be 9.99/1000 according to a population-based survey in southern Pakistan [4], and recent epidemiology data is unavailable [5]. About 80% of people living with epilepsy live in areas with limited resources, encountering significant barriers to health care services [6]. This makes it exceedingly challenging for them to adhere to daily self-management practices for their condition, including taking medications as directed, keeping consistent sleep patterns, engaging in regular physical activity, and effectively managing stress [7].

The evolution of mobile health (mHealth) apps has transformed them from basic information sources into advanced tools that can diagnose, monitor, and manage chronic neurological diseases [8,9]. mHealth apps, which are within the domain of eHealth and are defined as the usage of mobile and wireless communication technology to enhance health care delivery, outcomes, and research [10,11]. mHealth apps can provide substantial support to people living with epilepsy, encompassing medication reminders and alerts, educational resources, personalized feedback, cost management, medication education, and support [12-14].

In recent years, numerous mobile phone apps have been developed and effectively used to enhance the self-management of individuals with diabetes mellitus, hypertension, and various other conditions [15]. Applications for self-management of epilepsy have been implemented in advanced nations like Australia and the United States [16]. Although Pakistan claims over 90% mobile network coverage, this statistic pertains mostly to basic signal availability and does not reflect smartphone accessibility [17]. National Information and Communication Technologies studies indicate smartphone ownership at approximately 50%-60%, with diminished adoption in rural regions [18]. Digital literacy barriers, such as literacy levels, gender gaps, and socioeconomic disparities, strongly influence whether mobile coverage translates into meaningful use of health applications [19]. These factors form critical preconditions for the feasibility of epilepsy-focused mHealth solutions. Moreover, the extent to which patients are willing to use mHealth apps also affects their widespread adoption and successful integration into current health care systems [20,21]. Shreds of evidence [22,23] have reported that the sustainability of mHealth apps depends on feasibility studies. In LMICs, there is insufficient study investigating public perceptions of mHealth apps [24].

Several international studies have been conducted to evaluate the feasibility and acceptability of these mHealth apps. For example, in Uganda, Tumuhimbise and colleagues [25] found the use of mHealth apps feasible among health care workers to increase patient-based care to control tuberculosis. A pilot intervention study in British Columbia demonstrated the feasibility of the mHealth app among patients, which used automated SMS text messaging from a clinic's computer system [26]. An observational research in South Africa found epileptic-specific mHealth apps feasible among children with refractory epilepsy [27]. These studies collectively indicate the potential of mobile technologies to improve self-management. Despite promising international feasibility evidence, there is a lack of empirical evidence from Pakistan on perception, feasibility, and willingness to use epilepsy-focused mHealth apps. Nonetheless, these contexts are indicative of high- or middle-income environments with a stronger digital infrastructure, which restricts their applicability to Pakistan [28-30]. In Pakistan, the disparities in smartphone ownership, digital literacy, and cultural acceptance of health technologies indicate that feasibility has yet to be examined [8,31]. The absence of context-specific evidence highlights the significance of this study.

We contextualize feasibility within broader access and literacy constraints, and mobile coverage alone is not sufficient. Therefore, this study sought to investigate the feasibility, awareness, and perceptions of mHealth apps among people living with epilepsy and to analyze the factors that may affect this population’s willingness to adopt mHealth apps for epilepsy management.

## Methods

### Overview

The study was reported by following the STROBE (Strengthening the Reporting of Observational Studies in Epidemiology) checklist, adhering to the principles established by the Equator network [32]. The STROBE checklist has been attached in Multimedia Appendix 1.

### Study Design and Study Setting

This hospital-based quantitative research follows a cross-sectional analytical study, which was conducted among people living with epilepsy from March to July 2024 using a self-administered questionnaire.

The study was conducted at the Neurology department of the Pakistan Institute of Medical Sciences (PIMS), Islamabad [33]. The institute is the largest hospital in Islamabad, with a specialized 24-bed care unit for people living with epilepsy, equipped with the latest neurodiagnostic facilities. The institute is a well-known government hospital representing Pakistan due to its centrality. The Outpatient Department (OPD) serves 80-100 people living with epilepsy, providing free-of-cost treatment and diagnostic services [34].

### Study Population

The study participants’ having sex (male and female), consisted of people living with epilepsy aged 18 years and older who were able to provide informed consent, had a clinical diagnosis, and had been undergoing treatment for more than 6 months. People living with epilepsy with limited verbal communication, aged more than 65 years, experiencing hearing or cognitive impairments, and with a treatment history of less than 3 months were excluded from the study. Furthermore, the study excluded people living with epilepsy who were unable to complete the questionnaire.

### Sample Size and Sampling Technique

The online Rao soft calculator [35] was used to determine the sample size, incorporating a 5% margin of error, a 95% CI, and a 50% response rate. The calculation determined that a total sample size of 379 people was required. To secure a sufficient respondent pool, 427 people living with epilepsy were solicited, resulting in 406 being incorporated into the final analysis. A nonprobability sampling method, specifically convenience sampling, was used due to constraints in gathering data from a certain group of people living with epilepsy at specified times and locations.

### Questionnaire Development and Data Collection Procedure

The data-gathering questionnaire was developed in English based on a relevant scientific literature review [36-41]. A preliminary evaluation was performed before commencing the data collection process to assess the content validity, face validity, and internal consistency of the questionnaire. Five pharmacy practice professionals, in conjunction with the head of the Neurology department at PIMS, conducted a comprehensive assessment of the questionnaire to ensure face and content validity. The feedback obtained was used to enhance the questionnaire, ensuring its conformity with the study’s aims.

A pilot study with 28 participants was done to ascertain the clarity, comprehensibility, and alignment of the questions with the study’s aims. The pilot study results exhibited clarity, readability, and applicability. The feedback from the pilot study was integrated, resulting in the development of the final improved questionnaire before full implementation. Cronbach ⍺ value of the finalized questionnaire was determined to be 0.723. The pilot study was excluded from the final analysis.

The self-administered questionnaire was comprised of 5 sections containing 33 questions. Throughout the development process, these items were allocated into 5 segments, with certain items dependent on the responses to prior items. The initial segment sought to gather data regarding the demographic attributes, educational background, and treatment records of the participants. The second section aimed to collect data regarding the people living with epilepsy awareness of mHealth apps. In the third section, the people living with epilepsy evaluated their perceptions of mHealth apps using a 5-point Likert scale. The fourth section sought to assess people living with epilepsy comfort and practicality with mHealth apps. The final fifth component aimed to collect data on the willingness, level of agreement, or degree of concern regarding the use of mHealth apps for disease management. BBZ also developed the Urdu-translated version of the questionnaire for Urdu-speaking participants by using the World Health Organization’s translation guidelines [42]. Pharmacy practice specialists and neurologists meticulously evaluated the translated versions for clarity and contextual appropriateness.

For recruitment, BBZ was assigned a room within the neurology OPD by the department head in PIMS. After people living with epilepsy had completed their routine consultation with the attending neurologist, those meeting the predefined inclusion and exclusion criteria were approached by BBZ. The study objectives were explained, and informed written consent was obtained. The structured questionnaire was then administered. Each questionnaire lasted approximately 20-25 minutes. In total, 427 individuals were invited to participate, of whom 406 provided complete responses and were included in the final analysis. Twenty-one incomplete questionnaires were excluded, resulting in a study response rate of 95.1%.

### Statistical Analysis

The questionnaire was checked for completeness, and data were entered and analyzed using SPSS (IBM Corp). Descriptive statistics were applied to estimate the frequencies and percentages. The categorical data were presented as percentages and frequencies, while means and SDs were computed for continuous variables. The Mann-Whitney *U* test and Kruskal-Wallis test, when appropriate, were used for unadjusted comparisons. To examine the relationship between awareness, perception, feasibility, and willingness, the Spearman rank correlation coefficient was used. The independent relationships between willingness scores and clinical, sociodemographic, and attitudinal characteristics were investigated using multivariable linear regression analysis. Age, gender, type of residence, education level, employment status, family income, duration of treatment, daily pill burden, prescribed antiepileptic medication, awareness, perception, and feasibility scores were all considered independent variables. Variables were selected based on their theoretical relevance and significant associations identified through univariate analysis. Model assumptions, such as linearity, residual normality, homoscedasticity, and multicollinearity, were assessed prior to analysis and were determined to be adequate. A 95% CI and a *P* value of .05 were considered statistically significant. Due to the cross-sectional design of the study, these analyses were interpreted as exploratory associations rather than causal inferences.

### Ethical Considerations

The study was conducted in adherence with the guidelines outlined in the Declaration of Helsinki [43]. The Research Ethics Committee of Riphah Institute of Pharmaceutical Sciences, Riphah International University, Islamabad, Pakistan (reference number REC/RIPS/30), and Shaheed Zulfiqar Ali Bhutto University, Islamabad, Pakistan (reference number F.1-1/2015/ERB/SZABMU/1251), approved the research study. Participants were informed about the purpose of the research and its benefit to the population, and informed consent forms were obtained for each participant. The data confidentiality was also assured. No financial or nonfinancial compensation was provided to study participants.

## Results

### Sociodemographic and Clinical Characteristics of Participants

The predominant study population consists of males (218/406, 53.7%), married individuals (262/406, 64.5%), and middle-aged individuals of 3-45 years (152/406, 30%) of age, with a majority adhering to the Muslim faith (362/406, 89.2%). The majority of the people living with epilepsy underwent treatment for 2-3 years (199/406, 49%) and experienced a daily pill burden of 1-5 pills (257/406, 63.3%), and the predominantly prescribed antiepileptic medicine was valproic acid (297/406, 73.2%). The comprehensive demographic and clinical attributes of the study population are delineated in Table 1.

**Table 1:** Sociodemographic and clinical characteristics of the participants.

Characteristics			Participants, n (%)
**Age range (years)**			
	18-30	122 (30)	
	31-45	152 (37.4)	
	46-60	85 (20.9)	
	≥61	47 (11.6)	
**Sex**			
	Male	218 (53.7)	
	Female	188 (46.3)	
**Religion**			
	Muslim	362 (89.2)	
	Non- Muslim	44 (10.8)	
**Marital status**			
	Married	262 (64.5)	
	Single	121 (29.8)	
	Divorced	10 (2.5)	
	Widowed	13 (3.2)	
**Type of residence**			
	Rural	139 (34.2)	
	Urban	267 (65.8)	
**Educational background**			
	Uneducated	47 (11.6)	
	Primary or secondary	65 (16)	
	College	226 (55.7)	
	Bachelors or postgraduation	68 (16.7)	
**Employment status**			
	Employed	206 (50.7)	
	Self-employed	76 (18.7)	
	Retired	12 (3)	
	Unemployed	61 (15)	
	Housewife	51 (12.6)	
**Family income (US $)**			
	≤177	223 (54.9)	
	≥177	167 (41.1)	
	≥354	16 (3.9)	
**Duration of treatment**			
	>6 months	125 (30.8)	
	2-3 years	199 (49)	
	≥4 years	82 (20.2)	
**Daily pill burden**			
	1-5	257 (63.3)	
	6-10	121 (29.8)	
	>10	28 (6.9)	
**Medicine prescribes (antiepileptics)**			
	Valproic acid	297 (73.2)	
	Levetiracetam	44 (10.8)	
	Carbamazepine	30 (7.4)	
	Clonazepam	19 (4.7)	
	Lamotrigine	16 (3.9)	

### Correlation Among Awareness, Perception, Feasibility, and Willingness About mHealth Apps

There is a very weak positive correlation between awareness and perception (ρ=0.012; *P*=.08). This suggests that as the awareness scores increase, perception scores decrease slightly. Awareness shows a negative correlation with feasibility (ρ=–0.124; *P*=.01), which indicates that increased awareness toward mHealth apps is associated with increased feasibility of using mHealth apps. Awareness is weakly and positively correlated with willingness (ρ=0.013; *P*=.07); however, the correlation is not statistically significant. Perception shows a positive but weak correlation with feasibility (ρ=0.189; *P*˂.001), and this correlation is statistically significant.

### Awareness About the mHealth Apps

The majority of the participants, 86.2% (350/406), reported that they have heard about mHealth apps used for epilepsy management. Similarly, 86.2% (350/406) of participants were aware of the potential benefits of mHealth apps. About 72.9% (296/406) of respondents used mHealth apps and found them helpful. In contrast, 57.1% (232/406) of participants have not entered information into mobile apps related to disease and medication, highlighting a deficiency in the use of technology for managing chronic diseases (Table 2).

**Table 2:** Awareness about the mobile health apps.

Statements	Yes, n (%)	No, n (%)	I don’t know, n (%)
Have you ever heard of mobile health apps for epilepsy management?	350 (86.2)	49 (12.1)	7 (1.7)
Are you familiar with specific mobile apps for tracking seizures, medications, or other aspects of epilepsy management?	343 (84.5)	39 (9.6)	24 (5.9)
Have you ever used a mobile health app to monitor your epilepsy symptoms, medication, or overall health?	313 (77.1)	58 (14.3)	35 (8.6)
If you have used any mobile health apps for epilepsy, did you find the app features helpful?	296 (72.9)	92 (22.7)	18 (4.4)
Are you aware of the potential benefits of using mobile health apps for medication adherence?	350 (86.2)	36 (8.9)	20 (4.9)
Have you ever entered information into a mobile appn about your disease, medications, and diet?	133 (32.8)	232 (57.1)	41 (10.1)

### Perception, Feasibility, and Willingness About mHealth Apps

Overall, 69.2% (282/406) of participants were not open to using mHealth apps as an additional tool for epilepsy management. More than half of the participants (317/406, 78.1%) perceived that mHealth apps are not a valuable addition to their epilepsy management toolkit. In total, 75.9% (308/406) of the participants reported that mHealth apps can make medical care cheaper and faster. Meanwhile, 77.3% (314/406) reported that mHealth apps can save them from transportation costs, offering them significant financial relief. Around 69% (280/406) of participants had privacy concerns regarding medical information security offered by mHealth apps.

A total of 82.8% (336/406) of respondents indicated challenges in the usability of mHealth apps, and due to this reason, more than half participants (317/406, 78.1%) were not comfortable while using the mHealth apps on smartphones or tablets. Similarly, 79.3% (322/406) of participants reported that they did not find the app feasible or convenient for managing their disease. Overall, 36.7% (149/406) were not interested, and 73.4% (298/406) did not prefer mHealth apps for epilepsy management. Detailed perception, feasibility, and willingness about mHealth apps can be seen in Tables 3-5.

**Table 3:** Perception response of participants toward mobile health apps.

Statement and response	Strongly disagree, n (%)	Disagree, n (%)	Neutral, n (%)	Agree, n (%)	Strongly agree, n (%)
Are you open to using mobile health apps as an additional tool for managing epilepsy?	281 (69.2)	59 (14.5)	15 (3.7)	28 (6.9)	23 (5.7)
Do you think a mobile health app is a valuable addition to your epilepsy management toolkit?	317 (78.1)	14 (3.4)	35 (8.6)	31 (7.6)	9 (2.2)
Do you think mobile health apps make medical care cheaper and faster?	2 (0.5)	34 (8.4)	308 (75.9)	36 (8.9)	26 (6.4)
Do you think mobile health apps save money and transportation costs?	3 (0.7)	43 (10.6)	24 (5.9)	314 (77.3)	22 (5.4)
Do you think mobile health apps can improve and increase antiepileptic medication adherence?	18 (4.4)	294 (72.4)	44 (10.8)	39 (9.6)	11 (2.7)
Do you think mobile health apps increase communication with healthcare providers?	16 (3.9)	310 (76.4)	42 (10.3)	25 (6.2)	13 (3.2)
Do you think using mobile health apps to access health care offers great security?	25 (6.2)	25 (6.2)	280 (69.0)	68 (16.7)	8 (2)
Do you think it is an important tool that can be used to complement treatment strategies?	16 (3.9)	286 (70.4)	44 (10.8)	50 (12.3)	10 (2.5)

**Table 4:** Feasibility response of participants toward mobile health apps.

Statement and response			Participants, n (%)
**How** **comfortable are you in using smartphones or tablets to manage your epilepsy?**			
	Very uncomfortable	2 (0.5)	
	Uncomfortable	317 (78.1)	
	Neutral	53 (13.1)	
	Comfortable	18 (4.4)	
	Very comfortable	16 (3.9)	
**Do you think it is easy to handle a mobile health app?**			
	Strongly disagree	2 (0.5)	
	Disagree	5 (1.2)	
	Neutral	336 (82.8)	
	Agree	62 (15.3)	
	Strongly agree	1 (0.2)	
**Do you think it is convenient to use and share medical information on mobile health apps?**			
	Strongly disagree	2 (0.5)	
	Disagree	26 (6.4)	
	Neutral	322 (79.3)	
	Agree	53 (13.1)	
	Strongly agree	3 (0.7)	
**Would you prefer the mobile app to have medication reminders and tracking features?**			
	Strongly do not prefer	298 (73.4)	
	Do not prefer	44 (10.8)	
	Neutral	27 (6.7)	
	Prefer	17 (4.2)	
	Strongly prefer	20 (4.9)	

**Table 5:** Willingness response of participants toward mobile health apps.

Statement and response			Participants, n (%)
**I feel positive about using mobile health apps to manage my epilepsy condition**			
	Strongly disagree	11 (2.7)	
	Disagree	32 (7.9)	
	Neutral	117 (28.8)	
	Agree	142 (35)	
	Strongly agree	104 (25.6)	
**I am interested in using mobile health apps to monitor medication adherence**			
	Not interested at all	8 (2)	
	Not very interested	47 (11.6)	
	Neutral	114 (28.1)	
	Somewhat interested	149 (36.7)	
	Very interested	88 (21.7)	
**I am worried about my privacy and confidentiality if my health information is online**			
	Not worried at all	17 (4.2)	
	Not very worried	40 (9.9)	
	Neutral	122 (30)	
	Worried	146 (36)	
	Very worried	81 (20)	
**I prefer digital health tools over conventional hospital visits**			
	Strongly do not prefer	19 (4.7)	
	Do not prefer	48 (11.8)	
	Neutral	94 (23.2)	
	Prefer	149 (36.7)	
	Strongly prefer	96 (23.6)	

### Impact of Sociodemographic and Clinical Characteristics on Awareness, Perception, Feasibility, and Willingness About mHealth Apps

The median level of appropriate awareness among people living with epilepsy possessing a college education exceeded that of people living with epilepsy with primary or secondary education or those with bachelor’s or postgraduate degrees (*P*˂.001). The awareness of mHealth apps among people living with epilepsy was higher for the self-employed compared to retirees, the unemployed, employees, and housewives (*P*˂.001). Statistically significant differences were also found between awareness of mHealth apps and duration of treatment, which was higher in people living with epilepsy whose duration of treatment was ≤1 year as compared to 2-3 years or ≥4 years (*P*˂.001). The perception score was higher in people living with epilepsy aged 31-45 years as compared with those aged 18-30 years, 46-60 years, and ≥61 years (*P*=.04). Furthermore, the perception score was elevated in people living with epilepsy whose family income was ≥$354 compared to those with a family income of ≥$177 and ≤$177(*P*=.01). People living with epilepsy having a daily 6-10 pill burden have greater perception scores than those with a daily pill burden of 1-5 pills and ˃10 pills (*P*˂.001).

The feasibility score for using mHealth apps among people living with epilepsy with bachelor’s or postgraduate degrees surpassed that of people living with epilepsy with primary or secondary education, as well as those with college degrees and those who are uneducated (*P*˂.001). The feasibility score was also greater in people living with epilepsy having a family income of ≤$177 as compared to a family income of ≥$177 and ≥$354 (*P*≥.99). Moreover, people living with epilepsy having a treatment duration of ≥4 years have a higher feasibility score than those with ˂1 year and 2-3 years (*P*˂.001). The willingness score was greater among participants aged 31-45 years as compared to those aged 18-30 years, 46-60 years, and ≥61 years (*P*=.03). People living with epilepsy following the Muslim religion has a higher willingness toward mHealth apps as compared to non-Muslims (*P*˂.001). People living with epilepsy from urban residential areas exhibit a higher willingness score compared to those from rural locations (*P*≥.99). Comprehensive findings about the impact of demographic and clinical variables on the awareness, perception, feasibility, and willingness to adopt mHealth apps are provided in Table 6.

**Table 6:** Comparison of sociodemographic and clinical characteristics with awareness, perception, feasibility, and willingness of participants toward mobile health apps.

Characteristics			Participants, n (%)		Awareness						Perception							Feasibility							Willingness			
					Median (IQR)		*P* value			Median (IQR)			*P* value			Median (IQR)				*P* value			Median (IQR)				*P* value	
**Age (years)**								.04						.002							.002							.03
	18-30	122 (30)		5 (3-5)					18 (18-19.25)						9 (9-11)							15 (12-17)						
	31-45	152 (37.4)		5 (4-6)					19 (18-23)						9 (9-9)							15 (12-18)						
	46-60	85 (20.9)		5 (3-5)					18 (18-20)						9 (9-10)							15 (14-17)						
	≥61	47 (11.6)		5 (4-6)					18 (18-20)						9 (9-11)							13 (11-16)						
**Sex**								.65						<.001							.18							.80
	Male	218 (53.7)		5 (4-6)					19 (18-22)						9 (9-10)							1 5 (12-17)						
	Female	188 (46.3)		5 (3-5)					18 (18-19.75)						9 (9-10)							15 (12-17)						
**Religion**								.05						.09							.56							<.001
	Muslim	362 (89.2)		5 (4-5)					18 (18-21)						9 (9-10)							15 (12-17)						
	Non-Muslim	44 (10.8)		4 (2.25-5)					18 (17.25-19)						9 (9-11)							12.50 (10-15)						
**Marital status**								.09						.03							.12							.10
	Married	262 (64.5)		5 (3-5)					19 (18-21)						9 (9-10)							15 (12-17)						
	Single	121 (29.8)		5 (4-5)					18 (18-20)						9 (9-10)							15 (12-17)						
	Divorced	10 (2.5)		5 (2.75-5)					18 (17.75-24.50)						10.50 (9-15.25)							13.50 (9.50-14)						
	Widowed	13 (3.2)		5 (5-6)					18 (17-18)						9 (9-10)							13 (12-14)						
**Type of residence**								.06						.39							.44							.006
	Rural	139 (34.2)		5 (4-6)					19 (18-20)						9 (9-10)							14 (12-16)						
	Urban	267 (65.8)		5 (3-5)					18 (18-20)						9 (9-10)							15 (12-18)						
**Educational background**								.001						.17							<.001							<.001
	Uneducated	47 (11.6)		5 (3-5)					19 (18-21)						9 (9-10)							15 (12-16)						
	Primary or Secondary	65 (16)		4 (3-5)					18 (18-20)						10 (9-12.50)							14 (11-16)						
	College	226 (55.7)		5 (4-6)					18.50 (18-21)						9 (9-9)							16 (14-19)						
	Bachelors or postgraduation	68 (16.7)		5 (3.25-6)					18 (18-19)						9 (9-14.75)							12 (10-15)						
**Employment status**								<.001						.001							.74							.01
	Employed	206 (50.7)		5 (3-5)					18 (18-20)						9 (9-10)							16 (13-18)						
	Self-employed	76 (18.7)		5 (5-6)					19 (18-21.75)						9 (9-10)							13.50 (11-16)						
	Retired	12 (3)		5 (4-6)					18 (16.25-24.75)						9.50 (9-13)							13.50 (12- 15.75)						
	Unemployed	61 (15)		4 (3-5)					19 (18-23)						9 (9-11.50)							15 (12-17)						
	Housewife	51 (12.6)		4 (3-5)					18 (18-18)						9 (9-9)							15 (13-17)						
**Family income (US dollars)**								.19						.02							.002							.002
	≤$177	223 (54.9)		5 (3-5)					19 (18-22)						9 (9-11)							15 (13-17)						
	≥$177	167 (41.1)		5 (3-5)					18 (18-20)						9 (9-9)							14 (12-16)						
	≥$354	16 (3.9)		5 (5-6)					18 (18-23.50)						9 (9-9)							16.50 (15.25-19)						
**Duration of treatment (years)**								.001						.45							<.001							<.001
	≤1	125 (30.8)		5 (4-6)					18 (18-21)						9 (9-9)							15 (13-17)						
	2-3	199 (49)		5 (3-5)					18 (18-20)						9 (9-10)							16 (13-19)						
	≥4	82 (20.2)		4.50 (3-5)					18.50 (17-20.25)						10.50 (9-13.25)							12 (10-14)						
**Daily pill burden**								<.001						<.001							.41							.95
	1-5	257 (63.3)		5 (3-5)					18 (18-20)						9 (9-10)							15 (12-17)						
	6-10	121 (29.8)		5 (4-6)					19 (18-24.50)						9 (9-10.50)							15 (12-17)						
	>10	28 (6.9)		4 (3-5)					19 (17.25-21.75)						9 (9-10.75)							14.50 (12-19)						
**Medicine prescribes (antiepileptic)**								.10						.22							<.001							<.001
	Valproic acid	297 (73.2)		5 (4-6)					18 (18-20)						9 (9-10)							15 (13-18)						
	Levetiracetam	44 (10.8)		4 (3-5)					18 (18-22)						9 (9-10)							15 (12-17)						
	Carbamazepine	30 (7.4)		4 (3.75-5)					18 (18-19)						9 (9-10)							12 (11-14.25)						
	Clonazepam	19 (4.7)		4 (3-5)					19 (16-23)						11 (10-16)							12 (10-14)						
	Lamotrigine	16 (3.9)		5 (3.25-6)					18 (18-19)						9 (9-11)							13 (9.25-14.75)						

### Factors Associated With Participants’ Willingness to Use mHealth Apps

Multivariable linear regression analysis showed that the willingness to use mHealth apps differed among participants with varying educational backgrounds, residences, and family incomes. Rural resident participants have lower willingness than urban residents, with a *P* value of .05, which is borderline statistically significant. Participants having a college educational background have a significantly higher willingness (*P*˂.001) as compared to a bachelor’s or postgraduate degree. There was no statistically significant effect for uneducated or primary or secondary educated individuals. People living with epilepsy whose family income is ≥$177 show significantly lower willingness (*P*=.01). People living with epilepsy having a treatment duration of ≤1 year (*P*=.001) and 2-3 years (*P*˂.001) show significantly higher willingness as compared to those on treatment for ≥4 years. Other demographic and clinical factors, and awareness, perception, and feasibility scores do not show statistically significant influence. The findings from the multivariable linear regression analysis are summarized in Table 7.

**Table 7:** Multivariable linear regression analysis of the factors influencing the willingness of participants to use mobile health apps.

Characteristics				Participants, n (%)		Unstandardized coefficients, β (95% CI)		Standardized coefficients (β)		*P* value			
**Age range (years)**													
		18-30		122 (30)		0.446 (–0.81 to 1.70)		0.058		.49			
		31-45		152 (37.4)		–0.361 (–1.62 to 0.89)		–0.049		.57			
		46-60		85 (20.9)		0.738 (–0.58 to 2.06)		0.085		.28			
		≥61		47 (11.6)		Reference		—^a^		—			
**Sex**													
		Male		218 (53.7)		–0.025 (–0.75 to 0.70)		–0.004		.95			
		Female		188 (46.3)		Reference		—		—			
**Religion**													
		Muslim		362 (89.2)		0.275 (–0.83 to 1.38)		0.024		.63			
		Non-Muslim		44 (10.8)		Reference		—		—			
**Marital status**													
		Married		262 (64.5)		0.521 (–1.61 to 2.65)		0.070		.63			
		Single		121 (29.8)		0.155 (–2.02 to 2.34)		0.020		.89			
		Divorced		10 (2.5)		–0.374 (–3.22 to 2.47)		–0.016		.80			
		Widowed		13 (3.2)		Reference		—		—			
**Type of residence**													
		Rural		139 (34.2)		–0.729 (–1.47 to 0.01)		–0.098		.05			
		Urban		267 (65.8)		Reference		—		—			
**Educational background**													
		Uneducated		47 (11.6)		0.889 (–0.38 to 2.16)		0.080		.17			
	Primary or Secondary		65 (16)		0.530 (–0.69 to 1.75)		0.055		.39				
	College		226 (55.7)		2.27 (1.23-3.30)		0.319		<.001				
	Bachelor’s or postgraduation		68 (16.7)		Reference		—		—				
**Employment status**													
		Employed		206 (50.7)		0.678 (–0.56 to 1.91)		0.096		.28			
		Self-employed		76 (18.7)		–0.426 (–1.76 to 0.91)		–0.047		.53			
		Retired		12 (3)		–0.143 (–2.44 to 2.15)		–0.007		.90			
		Unemployed		61 (15)		–0.008 (–1.42 to 1.41)		–0.001		.99			
		Housewife		51 (12.6)		Reference		—		—			
**Family income (US dollars)**													
		≤177		223 (54.9)		–1.648 (–3.36 to 0.07)		–0.232		.06			
		≥177		167 (41.1)		–2.186 (–3.92 to –0.44)		–0.304		.01			
		≥354		16 (3.9)		Reference		—		—			
**Duration of treatment**													
		≤1 year		125 (30.8)		1.964 (0.83-3.09)		0.257		.001			
		2-3 years		199 (49)		2.203 (1.20-3.19)		0.311		<.001			
		≥4 years		82 (20.2)		Reference		—		—			
**Daily pill burden**													
		1-5		257 (63.3)		0.023 (–1.34 to 1.38)		0.003		.97			
		6-10		121 (29.8)		0.260 (–1.16 to 1.68)		0.034		.72			
		>10		28 (6.9)		Reference		—		—			
**Medicine prescribes (antiepileptics)**													
		Valproic acid		297 (73.2)		1.381 (–0.35 to 3.11)		0.173		.12			
		Levetiracetam		44 (10.8)		1.778 (–0.14 to 3.70)		0.156		.07			
		Carbamazepine		30 (7.4)		–0.265 (–2.27 to 1.74)		–0.020		.80			
		Clonazepam		19 (4.7)		0.616 (–1.54 to 2.77)		0.037		.58			
		Lamotrigine		16 (3.9)		Reference		—		—			
**Awareness score**													
	Continuous		406 (100)		–0.058 (–0.31 to 0.20)		–0.022		.66				
**Perception score**													
		Continuous		406 (100)		–0.041 (–0.11 to 0.03)		–0.053		.29			
**Feasibility score**													
		Continuous		406 (100)		–0.039 (–0.20 to 0.12)		–0.025		.63			

^a^Not applicable.

## Discussion

### Principal Findings

This study found that while most participants have heard about mHealth apps used for epilepsy management, the majority expressed concerns about privacy and security associated with these apps. More than half of the participants felt uncomfortable while using them due to challenges faced in the usability of mHealth apps. Similarly, the majority of participants reported that mHealth apps were not a valuable addition to their epilepsy management toolkit. In contrast, the study findings indicate that middle-aged urban residents with higher educational attainment and extended durations of epilepsy treatment exhibit a greater self-reported willingness to embrace mHealth apps as a digital health tool. This study highlights that awareness of mHealth apps and trial use do not necessarily translate into active, disease-focused usage.

The study findings indicated that participants with a high level of education were more likely to use mHealth apps for epilepsy management. Our findings are in line with the results of previous studies [44,45]. A considerable number of individuals with lower levels of education face significant health care needs; yet, they frequently struggle to comprehend the information provided on mHealth apps [46]. Ongoing endeavors to guarantee that mHealth apps are user-friendly and that health and treatment information is accessible, comprehensible, and retrievable are essential.

Participants living in rural regions exhibit a reduced willingness to engage with mHealth apps. This result supports earlier findings [47,48] indicating that the urban population exhibited a greater level of agreement regarding the use of mHealth apps. In resource-limited settings, traditional forms of health care provision are used more than advanced health care services [49]. The implementation of mHealth apps is difficult in LMICs due to the perpetuation of digital divides [50]. Therefore, by increasing the accessible treatment facilities and addressing the digital divides, we may assist the rural population affected by epilepsy in benefiting from these apps.

In total, 50% of the participants in the study expressed apprehension about sharing their medical information online, as this heightens their privacy concerns over the usage of mHealth apps that monitor their symptoms and facilitate illness treatment, which is compatible with the study conducted in Germany [51]. The transmission of patient information via digital tools presents legal concerns. Encryption must be adequate to comply with the Health Insurance Portability and Accountability Act (HIPAA) standards, ensuring patient confidentiality and the security of personal data [52]. Awareness about data privacy and the availability of medical information of people living with epilepsy should be provided to people living with epilepsy visiting hospitals for follow-ups, and privacy-preserving edge federated learning should be incorporated in mobile health systems [53].

In the study, we found that people living with epilepsy have a negative perception regarding the use of mHealth apps for epilepsy management. The negative perception surrounding the digitalization of the health care system arises from various cultural, systematic, and technological barriers, including stigma and inadequate digital health literacy [54]. Previous research [55] indicates the disparity between the creation and implementation of mHealth apps and the patients for whom they are designed. We recommend that the government and health care institutions take proactive steps to establish a digital literacy program aimed at educating people living with epilepsy and their caregivers on how to effectively access and navigate the mHealth apps, ultimately leading to improved treatment outcomes.

A recently published systematic review [8] indicates that people living with epilepsy are generally cognizant of mHealth apps, but there is a deficiency in comprehension of the app features and benefits that could facilitate timely disease management. This lack of understanding may impede the usage of these apps and exacerbate the atypical concerns of people living with epilepsy, which is consistent with previous research findings [56-58]. Awareness and practical implementation of mHealth app features for epilepsy management should be initiated in government hospitals to alleviate the disease burden on the impoverished population affected by epilepsy in Pakistan.

We also found that occupation correlates with the viability of using mHealth apps among people living with epilepsy. This study indicated that self-employed and employed participants were inclined to use mHealth apps. This is attributed to the high educational and socioeconomic status that facilitates their access to mHealth apps [59]. In contrast, the unemployed participants demonstrated a lower feasibility for using the mHealth app. This finding was similar to a multiethnic study [60] in the United States, which indicates that individuals from lower socioeconomic backgrounds had limited access to advanced treatment options.

A significant portion of the participants expressed discomfort and demonstrated a decreased preference for mHealth apps that include medication reminders and tracking functionalities. Previous studies [61,62] have demonstrated the significance of using mHealth apps in the management of chronic diseases. A key difficulty for 50%-60% of chronically ill persons is medication adherence [63]. mHealth apps appear to be a promising strategy for addressing issues related to medication nonadherence and enhancing treatment outcomes [64]. To improve medication adherence and management in people living with epilepsy, it is crucial to offer guidance by health care providers on mHealth apps for those receiving care in hospitals with suboptimal treatment outcomes for epilepsy.

Our findings may further substantiate the necessity for rules and frameworks for the creation and categorization of epilepsy apps within the existing epilepsy app ecosystem for less formally educated individuals [65,66]. Moreover, studies reported [67,68] challenges in conveying referrals to health care facilities due to limited knowledge and obstacles encountered by the uneducated people living with epilepsy. The regular enhancement of digital literacy skills and education for uneducated people living with epilepsy in need is essential to optimize the efficacy of an mHealth intervention.

In this study, people living with epilepsy prefer the mHealth apps to the conventional hospital visits, as the majority of participants belong to rural areas. Frequent hospital visits can incur significant expenses, especially in rural regions, owing to transportation expenditures [69]. A comprehensive systematic literature review [70] substantiates that mHealth apps demonstrate significant potential in assisting individuals and health care systems amid the rising prevalence and financial burdens of chronic diseases globally by providing informational support and an easier way to contact health care providers [71]. Consequently, the incorporation of mHealth apps may assist people living with epilepsy in managing their condition by alleviating financial strains and treatment burden.

Our findings indicate a notable level of awareness regarding mHealth apps in Pakistan when compared to various other LMICs. However, engagement with functions tailored to specific diseases remains limited. This aligns with global observations, as documented in the systematic review by Gotlieb et al [8]. The analysis emphasizes that, although feasibility and acceptability appear encouraging, genuine adoption and sustained use are dependent upon factors such as usability, literacy, and support from the health system. This highlights the necessity for strategies that are specifically tailored to the context of Pakistan. The comparison of the study data with previously published research has been elaborated in Table 8.

**Table 8:** Comparison of the study data with previously published studies targeting mobile health apps.

Study	Country and setting	Population	Sample Size	Key variables measured	Awareness and use of mHealth apps	Active engagement	Key findings or predictors
Our study	Pakistan, [urban+rural] clinic	Adults with epilepsy on treatment	406	Awareness Perception Feasibility Willingnes Treatment Duration Education	77.1% reported having used mHealth apps	42.9% input disease or medication data	Higher self-reported willingness among middle-aged, urban, higher-educated participants
Patterson et al [72], 2022	Pakistan and United Kingdom	Patients with epilepsy referred via health workers	59	Diagnostic accuracy Referral timing	Not applicable (usage by health workers)	Not applicable	High appropriateness of referrals; timeliness improved
Alzamanan et al [73], 2024	Malaysia	Expert panel & literature review	-	Domains or features of self-management apps	Not applicable	Framework focus, not direct patient use	Identified key domains; usability rated highly among experts
Gotlieb et al [8], 2025	Multiple countries	Mixed epilepsy populations	20 studies in review	Effectiveness Feasibility Usability	Varied installation and uptake	Mixed engagement with disease-specific functions	Barriers: usability, literacy, infrastructure; predictors include usefulness, interface quality

### Limitations

The study presents multiple limitations. First, we used convenience sampling, which results in limited generalizability. Second, data were collected from PIMS, primarily frequented by people living with epilepsy with low educational backgrounds resulting from illiteracy; the participants exhibited a lack of focus on the study’s objectives. Third, in the questionnaire, certain questions were addressed on behalf of a people living with epilepsy. As a result, the responses may be influenced by the perspective of the person conducting the survey on behalf of a patient, leading to self-reported data. Fourth, this study is deficient in qualitative insights and factor analytic approaches. Moreover, the assessment of willingness relied on self-reporting instead of direct behavioral measures. While face and content validity were established, no formal psychometric validation (eg, factor analysis) of the feasibility, perception, and willingness scales was performed. Due to the execution of several subgroup comparisons without formal adjustment, there exists a risk of increased type I error; hence, these findings should be interpreted as exploratory and hypothesis-generating rather than confirmatory.

### Future Implications

In the future, policymakers, health care providers, and app developers need to work together in co-designing and implementing evidence-based strategies for the use of mHealth apps, while also ensuring the protection of privacy within these apps. Addressing public health concerns necessitates the implementation of digital literacy sessions and training programs. Future research may concentrate on longitudinal and interventional studies, examining the functionality and efficacy of these mHealth apps with larger sample sizes, alongside user observations and perspectives, particularly highlighting the dynamics of user-platform interaction, to evaluate the causal pathways of mHealth usage and the establishment of trustworthiness. Moreover, it is recommended to conduct psychometric validation of the feasibility, perception, and willingness scales using factor analytic methods. Objective behavioral metrics, such as app usage logs, need to be incorporated to validate self-reported willingness. Probability sampling across multiple centers should be used to improve representativeness.

### Conclusions

This study demonstrates that urban residents with higher socioeconomic status were more likely to use mHealth apps, although they raised concerns about privacy protection. Stakeholders should implement a robust privacy policy in these apps. Future research may focus on addressing the digital divides, along with cultural, systemic, and technological challenges, as informed by the identified impacting factors. To improve the comfort of people living with epilepsy with mHealth apps, it is crucial to implement training programs and digital literacy sessions for both users and health care providers, focused on enhancing the functionality and effectiveness of mHealth apps.

## Appendix

Multimedia Appendix 1 STROBE checklist.

## Abbreviations

HIPAA: Health Insurance Portability and Accountability Act
LMIC: low- and middle-income country
mHealth: mobile health
OPD: Outpatient department
PIMS: Pakistan Institute of Medical Sciences
STROBE: Strengthening the Reporting of Observational Studies in Epidemiology

## Data Availability

The datasets used and analyzed during the current study are available from the corresponding author upon reasonable request.
